# Recent Advances in Pathophysiology, Drug Development and Future Perspectives of SARS-CoV-2

**DOI:** 10.3389/fcell.2020.580202

**Published:** 2020-11-06

**Authors:** Desh Deepak Singh, Ihn Han, Eun-Ha Choi, Dharmendra K. Yadav

**Affiliations:** ^1^Amity Institute of Biotechnology, Amity University Rajasthan, Jaipur, India; ^2^Plasma Bioscience Research Center, Applied Plasma Medicine Center, Department of Electrical and Biological Physics, Kwangwoon University, Seoul, South Korea; ^3^College of Pharmacy, Gachon University of Medicine and Science, Incheon, South Korea

**Keywords:** SARS-CoV-2, pneumonia, pathophysiology, immune response, epidemiology, drug discovery

## Abstract

The coronavirus (SARS-CoV-2) pandemic is a rapidly transmitting and highly pathogenic disease. The spike protein of SARS-CoV-2 binds to the surface of angiotensin-converting enzyme-2 (ACE2) receptors along the upper respiratory tract and intestinal epithelial cells. SARS-CoV-2 patients develop acute respiratory distress, lymphocytic myocarditis, disseminated intravascular coagulation, lymphocytic infiltration, and other serious complications. A SARS-CoV-2 diagnosis is conducted using quantitative reverse-transcription PCR and computed tomography (CT) imaging. In addition, IgM or IgG antibodies are used to identify acute and convalescent illness. Recent clinical data have been generated by health workers and researchers and have shown that there is an urgent requirement in the effective clinical and treatment of patients, as well as other developments for dealing with SARS-CoV-2 infection. A broad spectrum of clinical trials of different vaccines and drug treatment has been evaluated for use against SARS-CoV-2. This review includes the emergence of SARS-CoV-2 pneumonia as a way to recognize and eliminate any barriers that affect rapid patient care and public health management against the SARS-CoV-2 epidemic based on the natural history of the disease, its transmission, pathogenesis, immune response, epidemiology, diagnosis, clinical presentation, possible treatment, drug and vaccine development, prevention, and future perspective.

## Introduction

**FIGURE 1:**
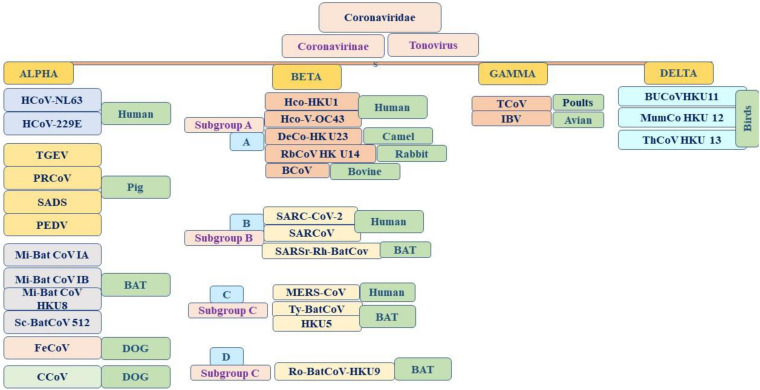
Emergence and taxonomy of coronavirus.

**FIGURE 2:**
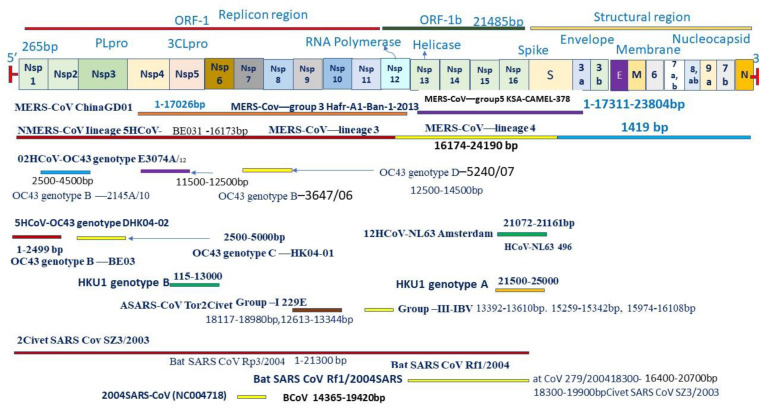
Genome and genetic shift at genomic site coronavirus with zoonotic origin.

**FIGURE 3:**
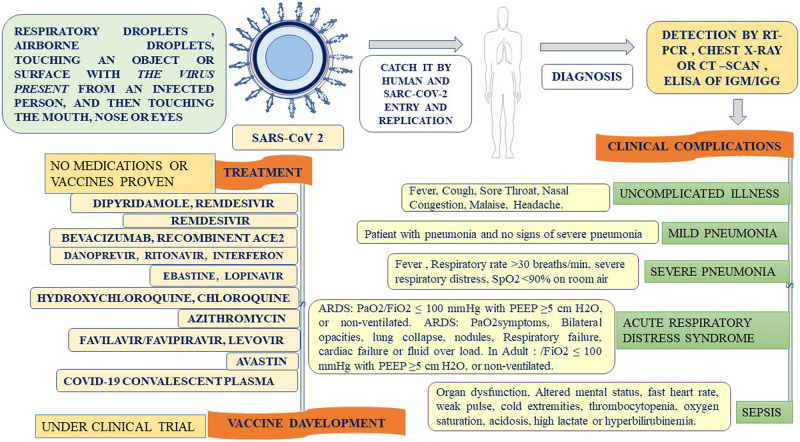
Flow diagram for diagnosis, clinical presentation, and treatment of SARS-CoV-2.

Coronaviruses are a group of large viruses with positive SS RNA, 160 nm in size, and belonging to the Nidovirales family (Schoeman and Fielding, 2019). The genome size of the SARS-CoV-2 virus is approximately 30 KB. The virion of SARS-CoV-2 is made up of genomic RNA with a structural protein, i.e., nucleocapsid (N) phosphoprotein, which encloses the nucleic acid, a glycoprotein membrane (M) embedded in the lipid bilayer, a spike glycoprotein (S) with petal-shaped peplomers and a small enveloped (E) protein and a polyadenylated tail, followed by 3′ ends (McBride et al., 2014; Schoeman and Fielding, 2019). Two-thirds of the SARS-CoV-2 genome made up of a polyprotein (pp1a and pp1b), which is produced after the cleavage of the cysteine protease (PLpro) and 3C–like serine protease (3CLpro), the resultant non-structured protein, and RNA–dependent RNA polymerase and helicase are synthesized (Narayanan et al., 2015). Six genera of Coronavirus have been discovered, namely, alpha corona, beta, gamma, delta, bafinivirus, and torovirus. Human coronavirus (HcoVs) belongs to the alpha and beta coronaviruses, genera 229 E and NL63 belong to the alpha coronavirus, and OC43 and HKU1 belong to the beta coronavirus (Figures 1, 2; Owusu et al., 2014). The main hosts and reservoir of these viruses are infected birds and mammals. Being hosts to the largest number of viral genotypes, bats have also become host to large numbers of coronaviruses, and their immune systems can suppress such viruses (Khan, 2013). However, they can transmit these viruses to birds and other mammals. Epidemics of coronaviruses generally occur when the virus transmits from one species to another, as shown in Figures 1, 2, which takes place when the viruses acquire mutations in proteins that allow them to bind to the cells of other animals and more easily infect the other cells in the new host. Human coronaviruses are causes of respiratory and gastrointestinal tract infections (Lau et al., 2010). Such viruses have been estimated to account for 5–10% of all adult upper respiratory tract infections such as the common cold, pneumonia, and acute respiratory distress syndrome (ARDS), and causing gastrointestinal infection (Ge et al., 2013; Khan, 2013). There are a wide variety of symptoms of coronavirus infection. Usually, mutations occur when a virus transfers from one species to another, and entirely new coronaviruses can develop through such mutations. Several outbreaks of new coronaviruses (Smith, 2006), the first being severing acute respiratory syndrome (SARS), occurred in 2002 to 2003, which was reported to have been a beta coronavirus from Guangdong province in China (Chan et al., 2003). There are different lineages of beta coronaviruses within SARS lineage B, and this particular virus has previously been transmitted from bats to civets to humans in an outbreak recorded from February to July 2003. In this outbreak, there were more than 8000 total recorded cases and 774 deaths with a fatality rate of 9.6% (Chan-Yeung and Xu, 2003). Another coronavirus outbreak of Middle East Respiratory Syndrome (MERS) occurred in 2012, as shown in Figures 1, 2 (Zaki et al., 2012), which reportedly began in Saudi Arabia and spread to several other countries. This was also a beta coronavirus and was transmitted from camels to humans (Lau et al., 2013) either by eating camels or drinking camel milk. More than 2400 cases and 858 deaths were recorded with a fatality rate of approximately 34.4%. The new coronavirus, 2019-nCoV (SARS-CoV-2) discovered in Wuhan, Hubei province of China in December 2019, the seventh coronavirus found to cause illness in humans. It is also a novel beta coronavirus and has many similarities with SARS (Chen N. et al., 2020; Huang C. et al., 2020). SARS-CoV-2 was first identified through a cluster of pneumonia cases in Wuhan, China On December 31, 2019, an infectious disease with a cluster of cases of pneumonia occurred in Wuhan, China, which was later identified as novel SARS-CoV-2 (Bernard Stoecklin et al., 2020). By January 31, 2020, 7818 cases had been confirmed in 19 countries, and on May 18, 2020, the WHO declared a coronavirus outbreak as a public health emergency, with 10500,639 cases, 510,736 deaths, and 5,727,094 recovered cases recorded in more than 200 countries. The virus is officially named SARS-CoV-2 because it is genetically extremely like SARS coronavirus, which was responsible for the outbreak in 2002 (Chen and Yu, 2020). It is believed that SARS-CoV-2 was transmitted from a bat to a Pangolin and finally to a human (Figure 2). A coronavirus found in a Pangolin showed a 96% genetic match with SARS-CoV-2. The spike protein of the coronavirus allows it to attach to the lining of the respiratory tract and damage the ciliated epithelial cells of the nasopharynx leading to viremia (Xu H. et al., 2020). Severe lung damage can cause ARDS and aseptic shock, which are the main causes of death for people infected and are more likely to occur in those over the age of 60, in smokers, and in people with previous medical conditions such as diabetes, Chronic Obstructive Pulmonary Disease (COPD), Cardiovascular Diseases (CVDs), hepatitis, hypertension, or pregnancy (Figure 3; Matthay et al., 2019). During this pandemic, one of the main concerns is the treatment options available. At present, we do not have any drugs approved for SARS-CoV-2 by the FDA, although there are certain drugs used by clinicians for patients with SARS-CoV-2 infection (Adnan, 2020), including redeliver, chloroquine, hydroxychloroquine, lopinavir, ritonavir, and tocilizumab (Abd El-Aziz and Stockand, 2020). None of these drugs are currently used as a prophylactic against SARS-CoV-2 but are administered after infection. It has been observed that chloroquine and hydroxychloroquine, which are antimalarial drugs, are more effective against SARS-CoV-2 (Kruse, 2020). Tocilizumab is most widely used by clinicians, and its IL-6 inhibitors lead to a reduction in cytokines and an acute phase retract. This drug is still under clinical trial for SARS-CoV-2 pneumonia. Lopinavir and ritonavir are two other drugs that have been used despite their limitations. Initially, they were used for HIV infection, and have shown to be active against SARS-CoV-2 in animal studies (Zhao, 2020). All these drugs are used by clinicians based on their severity, clinical complications, and special consent or ethical approval of the competent authority. Previous reviews on COVID-19 have been based on information regarding a specific problem. Limited reviews are available regarding the recent developments of SARS-CoV-2 pneumonia for recognizing and eliminating any barriers that affect the rapid patent care and public health management against the SARS-CoV-2 epidemic. This review aims to discuss recent advances in SARS-CoV-2 in terms of pathophysiology, epidemiology, clinical management, drug discovery, vaccine development, and prospects. This review includes the emergence of SARS-CoV-2, the natural history of the disease, and its transmission, pathogenesis, epidemiology, diagnosis, clinical presentation, possible treatment, drug and vaccine development, and prospects.

## Pathophysiology

**FIGURE 4:**
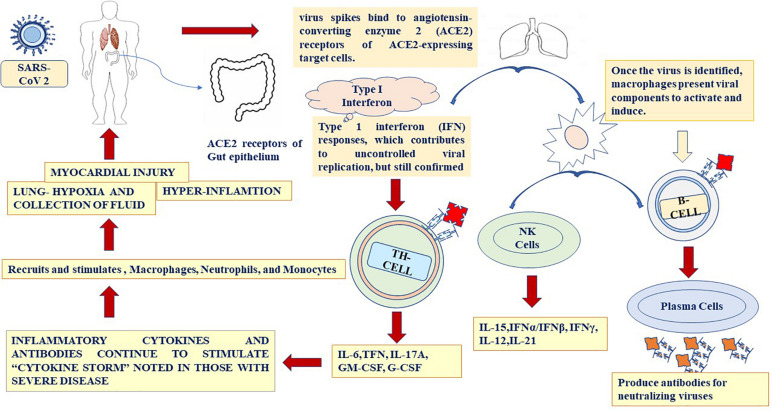
Immune response during SARS-CoV-2 infection.

SARS-CoV-2 is inhaled through respiratory droplets (>5 μm) in the air at up to <1 m (less than 3.3 feet) and within close contact (6 feet, or 1.8 m) (Cui et al., 2020). The virus is also inhaled by touching an object or surface with a virus present from an infected person, and then touching the mouth, nose, or eyes (Figure 4). SARS-CoV-2 causes ARDS and invades two types of cells in the lungs, namely, mucus-producing (Goblet) cells and ciliated cells, allowing it to reproduce (Singhal, 2020). Mucus keeps the lungs from drying out and protects them from pathogens. Celia pushes the mucus toward the exterior of the body, clearing debris from the lungs including viruses. Killer cells are the preferred host cells for SARS-CoV and are likely the preferred host cells for SARS-CoV-2 (Figure 3; Chen W. H. et al., 2020; Wang F. et al., 2020). Once a structural protein enters a cell, the genetic, viral material is copied. The invading virus blocks the synthesis of this viral genetic material. The invading virus blocks the synthesis of this viral genetic material (Tian et al., 2020). The virus enters the cell through fusion and endocytosis. The virus is partially decomposed and becomes insensitive to the neutralizing antibodies of the host (Belouzard et al., 2012). The host cell receptors, consider the virus to be a normal legend, leading to a receptor aggregation of the virus and the entry of the virus through the endocytosis process (Baig et al., 2020). Virus uncoating occurs inside the cytoplasm along with the release RNA inside the host cells, forming a polyprotein complex (Tian et al., 2020). These polyproteins are cleaved under the action of the protease into the RNA polymerase (Letko et al., 2020). Antigenomic RNA is transcribed from genomic RNA. The hallmark of coronavirus transcription is the production of multiple sub-genomic mRNAs, and during the replication process, two intermediates are formed: First, an intermediate genome forms an antigen and creates a new genome complex (Baig et al., 2020). RNA polymerase acts as an anti-genomic RNA to generate a positive genome strand and the m-RNA. Next, m-RNA acts on the rough endoplasmic reticulum of the host cells to produce a new viral genome (Baig et al., 2020; Letko et al., 2020). Common symptoms of SARS-CoV-2 include fever (>37.3°C), which may not be present in some cases, shortness of breath, coughing, sore throat, insomnia and/or ageusia, muscles ache, nausea/or vomiting, abdominal pain, headache, runny nose, and fatigue (Figure 3; Huang C. et al., 2020). Frequent symptoms at illness onset include fever (83–98%), dry cough (76–82%), and myalgia or fatigue (11–44%), and the incubation period varies from 5 to 14 days. Besides, 14% of patients were observed as having severe symptoms, and 5% were found to be critically ill (Zhou et al., 2020). According to the WHO, SARS-CoV-2 cases are steadily increasing throughout the world. Early reports suggested that illness severity is associated with age (>60 years old) and comorbidity (Liu B. et al., 2020). Clinical investigations of patients with SARS-CoV-2 have been based on early detection, identification, isolation, and employment of immediate prevention and control with supportive care of the patient through the management of ARDS and hypoxemic conditions with septic shock (Jiang et al., 2020). Special and urgent care is required for pregnant women. Patients are categorized into those with an uncomplicated illness, mild pneumonia, severe pneumonia, ARDS, sepsis, and septic shock (Du et al., 2020). When mucus-producing (goblet) cells and ciliated cells die owing to a collection of new SARS-CoV-2 material, they slough off into the airways, filling them with debris and fluid. Many of those infected experience pneumonia in both lungs (Li et al., 2020). The virus enters the immune system, and the immune cells recognize the virus flooding into the lungs (Jiang et al., 2020). The lung tissues then become inflamed during the normal immune response. The inflammatory process is highly regulated and is confined to the infected area (Liu H. et al., 2020). However, occasionally the immune system overreacts, resulting in damage to the healthy tissues. More cells then die and slough off, further clogging the lungs and worsening pneumonia (Feng Y. et al., 2020). As damage to the lungs increases, stage three begins, potentially resulting in respiratory failure, permanent lung damage, or death. Here, we can see the same lessons with SARS-CoV-2 as with SARS (Cowling and Leung, 2020). SARS create holes in the lungs, which have a honeycomb appearance. This is probably due to an over-reactive immune response, which affects healthy and infected tissue and generates scars that stiffen the lungs (Tan K.S. et al., 2020). Some patients with this condition may require ventilators to aid in breathing (Chen W. H. et al., 2020). The inflammation also results in alveoli that are more permeable. Alveoli are interfaces of gas exchange, where the lung replaces CO_2_ in the blood with fresh oxygen. The increased permeability causes fluid to leak into the lungs (Raoult et al., 2020; Zhou et al., 2020). This decreases the ability of the lungs to oxygenate the blood and in severe cases floods the lungs with fluid, preventing the patient from breathing properly and sometimes resulting in death (Zhang H.-T. et al., 2020). Venous thromboembolism is reduced using mechanical or pharmacological prophylaxis, and catheter-related bloodstream infections are removed by maintaining sterile and aseptic conditions, as well as the removal of the catheters daily (Goossens, 2015). Aseptic shock is the main cause of death for people with this infection and is more likely to occur in those over the age of 60, smokers, and people with previous medical conditions. Individuals with diabetes, cardiovascular disease, heart disease, hypertension, respiratory disease, pregnancy, or an immunocompromised state are high risk for clinical complications and mortality (Figure 3; Liu K. et al., 2020) An overreaction of the immune system causes another type of damage, namely, cytokines recruiting immune cells at the site of infection. Overproduction of cytokines can result in a cytokine storm and cause large-scale inflammation in the whole body of the patient (Puja et al., 2020). Therefore, blood vessels become more permeable and fluid seeps out. This situation makes it difficult for blood and oxygen to reach the patient’s body. As a result, multi-organ failure occurs in more severe cases of SARS-CoV-2.

## Immune Response

The site of the initial infection of SARS-CoV-2 is unknown, but the most common effect is damage to the respiratory system owing to the predominance of the angiotensin-converting enzyme 2 (ACE2) receptors in the epithelial lining (Elena et al., 2020). The gastrointestinal tract also has a significantly higher concentration of ACE2, but GI symptoms were seen more in SARS infections and have been quite rare in SARS-CoV-2 infections. The immune response in SARS-CoV-2 patients occurs in two phases: Initially, when the disease is moderate, the immune cells eliminate the virus by producing primary cytokines, which may be beneficial for eliminating the virus but results in inflammation. During the second phase of more severe cases, SARS-CoV-2 patients exhibit significantly high cytokine levels or hyper-inflammation, and in some cases, the lungs become damaged (Figure 4; Azin et al., 2020; Shih-Hwa et al., 2020). Patients in this stage develop ARDS, and worsening damage to the lungs is a major challenge. Some patients with severe respiratory symptoms experience a high viral load in the lungs, and further studies are required to understand the dynamics between viral load dynamics and effective immune response (Zhuobing et al., 2020). Extreme inflammation results in the release of large cytokines in the circulation, which start affecting the secondary organs, and the viral load begins to decrease. In addition to the lungs, the heart rate may also be affected by systemic inflammation during SARS-CoV-2 infection (Tian et al., 2020). Severe SARS-CoV-2 infections are associated with lymphocytopenia, which is a severe loss of lymphocytes in the bloodstream. Many cytokines are released in SARS-CoV-2 patients and cause a cytokine storm such as IL-6, IL-1, IL-2, IL-10, TNF-α, and IFN-, Increased level of IL-6 in the serum leads ARDS and adverse clinical outcome, such as respiratory failure (Tan K.S. et al., 2020; Tan L. et al., 2020). A high level of IL-6 can suppress normal cell activation (Azin et al., 2020). Thus, a cytokine storm may help with the suppression of T-cells in SARS-CoV-2 patients (Puja et al., 2020). Researchers have also suggested that a massive release of cytokines involves a rapid viral replication, leading to apoptosis. This increases the macrophages in the lungs, which in turn amplifies the inflammatory response (Figure 4). Specific cytokines are involved in the SARS-CoV-2 immune response. In severe cases of SARS-CoV-2 infection, increased levels of IL-6, IL-10, TFN-α, and the soluble IL-2 receptor have been found. In another study, elevated levels of IL-7, G-CSF, IP-10, MCP-1, and MIP-1α were found in patients with a severe SARS-CoV-2 infection (Liu H. et al., 2020). Patients experiencing a cytokine storm may have lung damage and multi-organ failure, with death occurring in some cases. Understanding the mechanism of the immune response by which the cytokine storm can be reduced is critical for the design of future therapies. Targeted immune cell-based therapies may prove beneficial because they focus on a specific cytokine, such as IL-6, IL-10, and TNF-α, without causing a widespread effect on the immune system (Figure 4; Ye et al., 2020). To fully understand the occurrence of a cytokine storm in SARS-CoV-2 patients, inflammation, acute phase, essential immune response, proinflammatory chemokines, immune cell-specific (including B cells, CD8, and natural killer cells), macrophage and microglia, and T-helper-cell cytokine panels need to be conducted on the serum or bronchoalveolar lavage fluid of COVID-19 patients (Zhang H.-T. et al., 2020).

## Diagnosis

Lab diagnosis of SARS-CoV-2 is shown in Table 1, where the chest X-ray shows patchy infiltrates, which may be peripheral or concentrated in the lower lung fields, or maybe interstitial infiltrates, an easily progressive problem involving diffusion and infiltration (Hong et al., 2020). To confirm the diagnosis, and (Real-Time Polymerase Chain Reaction) RT-PCR is conducted on a small amount of viral RNA specimen in the respiratory tract (sputum, swab, aspirate, or lavage), or plasma during the early disease stage (Corman et al., 2020). An RT-PCR is also conducted on the urine and stool at a later stage (Xu X. et al., 2020; Zhang J. J. et al., 2020). The cultivation of a coronavirus is extremely difficult; in fact, such viruses grow only in a human tracheal ring organ culture, except for SARS and MERS, which can easily grow on Vero cell lines (Coleman and Frieman, 2015). Antibodies appear after 1 month from infection, and ELISA and immunofluorescence antibody tests (IFATs) are used as a sociological investigation in the spread of the disease into the population. ELISA and immunofluorescence antibody test (IFAT) is used to detect for sociological investigation, which appears, after 1 month (Addie, 2020). Confirmed cases of SARS-CoV-2 have tested positive regardless of clinical symptoms. Probable cases are considered for those with inconclusive results or when testing cannot be performed under any circumstances (Jian-Min et al., 2020). Suspected cases are referred to as those patients with clinical symptoms, a history of travel to or residing in an infected area, or have had contact with someone with a confirmed or probable case during 14 days or more up to 28 days before the onset of symptoms or with someone with full clinical signs (Giuseppe and Rossella, 2020). Negative cases are considered individuals who have tested negative at least twice within at least a 48-h time interval with a confirmed or probable test result (Jian-Min et al., 2020). CRISPR (Clusters of Regularly Interspaced Short Palindromic Repeats) is a genome-editing tool. it helps in DNA sequences and functions of the gene (Singh et al., 2019; Verma et al., 2019). CRISPR has potential application in the prevention and diagnosis of disease. CRISPR -Cas 9 and its variants-based diagnostic under process. The expert from the United States published CRISPR based diagnosis method for SARS-CoV- in nature Biotechnology (Broughton et al., 2020).

**TABLE 1:** Types of diagnostic approaches in SARS-CoV-2.

**Types of diagnostic tests**	**Mechanism of detection**	**Source of samples**	**Result Interpretation**	**References**
Nucleic acid amplification	Real Time PCR and NGS sequencing by using gene specific primer such as N,S,E and RdRP genes two independent sequences need to be detected	Nasal Swab, throat Swab, Bronchoalveolar lavage, blood faces and endotracheal aspirate	SARS-CoV2 Infection	Corman et al., 2020
Antibody based immunoassay	SARS-CoV2 IgM and IgG antibodies detection by ELISA	Serum	Immunity/Overall infection	Addie et al., 2020
Antigen based immunoassay	SARS-CoV2 detection protein	Nasal Swab, throat Swab, Bronchoalveolar lavage, blood faces and endotracheal aspirate	Confirm current SARS-CoV2	Udugama et al., 2020
CT- Imaging	Clinical symptoms (Fever/Cough, epidemiological history imaging CT)	Radiological features	Trade to identify for further target	Zhuobing et al., 2020

## Treatment

The repurposing of drugs is an effective outcome-based treatment that also allows significantly enhancing the drug development process (Senanayake, 2020). Most treatment for SARS-CoV-2 has been based on supportive care such as the use of remdesivir, lopinavir/ritonavir, hydroxychloroquine, convalescent plasma, synthetic antibodies, interferons, low dose steroids, azithromycin, IL-6, IL-1 inhibitors, anti-TNF, and various other support treatments according to the clinical complications of the patients (Tables 2, 3; Wu D. et al., 2020). Ritonavir is an anti-HIV drug, and redeliver is an anti-viral drug previously used against the EBOLA virus. Remdesivir was given to the first United States patient with SARS-CoV-2, who began to improve the very next day (Eastman et al., 2020). Remdesivir is an intravenous drug, which has a broad antiviral activity, inhibiting viral replication through a pre-mature termination of the RNA transcription. This drug has shown an *in vitro* activity against SARS-CoV-2 and *in vitro* and *in vivo* activities against related beta coronaviruses, and the drug blocks RNA dependent polymerases (Cao Y.C. et al., 2020). There are various randomized trials underway to evaluate the efficacy of remdesivir for SARS-CoV-2 infection from moderate to severe cases (Beigel et al., 2020). A large-scale clinical trial is ongoing, and some of the results are shown in Tables 2, 3. Chloroquine and hydroxychloroquine are oral prescription drugs used for the treatment of malaria and various inflammatory clinical complications (Ferner and Aronson, 2020). Chloroquine has been used for malaria treatment and chemoprophylaxis, and hydroxychloroquine has been used for rheumatoid arthritis, systemic lupus erythematosus, and porphyria cutanea treatments (Alia and Grant-Kels, 2020). Both are drugs having *in vitro* activity against SARS-CoV-2 and other coronaviruses. Hydroxychloroquine has a higher potency against SARS-CoV-2 infection. Chloroquine has been reported to inhibit the *in vitro* activity of SARS-CoV-2 (Alia and Grant-Kels, 2020). Although published clinical data are limited, hydroxychloroquine in combination with azithromycin is more effective; however, validation is required and clinical trials are ongoing (Yazdany and Kim, 2020). The possibilities of drug toxicity and other clinical side effects should be considered before use. In May of 2020, the WHO stopped the clinical trial of hydroxychloroquine owing to various side effects. Italy, France, Belgium, and other countries also stopped using hydroxychloroquine (Meyerowitz et al., 2020). A study published in the New England Journal of Medicine reported that hydroxychloroquine is of no benefit in SARS-CoV-2 patients (Geleris et al., 2020). In April, the Food Drug Administration (FDA) issued an order to stop using hydroxychloroquine and chloroquine owing to potential cardiac problems (Boulware et al., 2020). Ibuprofen is anti-inflammatory and helps with breathing difficulty in patients. The clinical trial of Ibuprofen started on hospitalized patients with SARS-CoV-2 infections (Sodhi and Etminan, 2020). Monoclonal antibodies were isolated from surviving patients of SARS-CoV-2 and used for testing. During the first week of June in 2020, blood plasma was transferred in approximately 25 patients with SARS-CoV-2 infection at Methodist Hospital in Texas, United States (Salazar et al., 2020). Stem cell-based treatments are also under phase-II and -III clinical trials for use in SARS-CoV-2 infection (Raza and Khan, 2020). Immunosuppressant drugs are also under clinical trials, including baricitinib and an IL-6 inhibitor. In SARS-CoV-2 patients, the immune system becomes overactive and releases a cytokine storm. The FDA has therefore allowed the use of a device that filters cytokines from the blood of patients and improves the immune response. Even in various re-purposed and possibly novel therapies for SARS-CoV-2, developments in the clinical management of the patient is critical. In addition, there are still no future timelines for specific treatment options for SARS-CoV-2 (Zhao, 2020).

**TABLE 2:** SARS-CoV-2 candidate drug treatments in Phase III-IV trials.

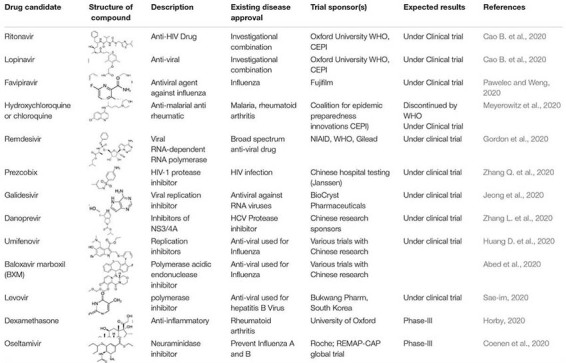

**TABLE 3:** Development of SARS-CoV-2 Immunoglobulin based treatments option.

**Leading candidate**	**Description**	**Trial sponsor(s)**	**Development of stage**
Convalescent plasma	Passively transfer antibodies (Immunoglobulin)	USA FDA -Sponsored	Phase-III
		Expanded access program coordinated by Mayo Clinic	
STI-5656 (Abivertinib)	Tyrosine kinase inhibitor	Sorrento Therapeutics	Phase-III
PRO 140 (Leronlimab)	Monoclonal antibody targeted against CCR5 receptor	CytoDyn Inc., United States	Phase-III
PTC299	Dihydroorotate dehydrogenase inhibitors	PTC therapeutics, Inc., South Plainfield, NJ, United States	Phase-III
CD24Fc	Immunomodulator (New drug)	Oncolmmune, Inc., United States	Phase-III
Lenzilumab	Chronic Myelomonocytic leukemia	Targets Colony Stimulating factors (CSF-2); Multiple countries	Phase-III
Tocilizumab	Immunosuppression	Roche holding AG, Basel, Switzerland	Phase-III
Sarilumab	Rheumatoid arthritis	Regeneron-Sanofi, United States	Phase-III
Ravulizumab	Compliment inhibitors	Alexion pharmaceuticals Inc	Phase-III
Losmapimod	MAPK as potent suppressors of DUX4 expression	Fulcrum Therapeutics, United States	Phase-III
Pepcid	H2 blocker	Yamanouchi Pharmaceutical Co., Merck, Japan	Phase-III
Mitigare (Colcrys)	Anti-inflammatory agent	Bill & Melinda Gates Foundation, United States	Phase-III

Clinicians and researchers are also looking for other options to target the replication or clinical manifestations of SARS-CoV-2. There is an urgent need to develop a sensitive and specific medicine for the various genotypes and stereotypes of SARS-CoV-2, such as cove 229E (HCoV-229E), OC43 (HCoV-OC43), NL63 (HCoV-NL63), and HKU1 (HCoV-HKU1), as shown in Figures 1, 2 (Bleibtreu et al., 2018). Preliminary investigations suggest that RNA a polymerase-based drug may inhibit the multiplication of HCoV infections (Adnan, 2020). There are various possible target RNA-dependent polymerase and helices for application as antiviral developers (Russell et al., 2020), which are produced by a replicase polyprotein and encoded by the ORF at the 5′end and cleaved by 3-C like proteases. The cleavage process occurring during membrane fusion leads to a syncytium formation. The binding of the amino-terminal receptor to the host cell surface leads to conformational changes in the carboxy-terminal, enabling fusion (Li et al., 2020). The structural spike glycoprotein of SARS-CoV-2 can also be targeted. Monoclonal antibodies have been developed using an angiotensin-converting enzyme 2 and are sensitive against SARS-CoV-2 at the site of the amino terminal-receptor binding domain and cause fusion with the host cells (Russell et al., 2020). After completion of SARS-CoV-2 multiplication cycles in the host, a version is released into the extracellular compartment through exocytosis, and viral multiplication cycles are repeated. The structural protein of SARS-CoV-2 may be targeted by small interfering RNA. Existing or earlier used drugs for HCoV-229E, HCoV-OC43, SARS-CoV, and the emerging MERS-curve can be further tested for cytopathies by applying a standard assay (Shen et al., 2019). A known pharmacokinetic and pharmacodynamic immunoglobulin approach may be employed by exploring the efficacy against SARS-CoV-2 (Shen et al., 2019; Li et al., 2020). In addition, a high throughput analysis of existing compounds can be conducted by using a database and expected antiviral activity along with their immunological and physiological efficacy. Genomic and biophysical based drugs can be developed including viral enzyme-based inhibitors. Moreover, all these drug options can be used with patients of SARS-CoV-2.

## Inhibitor of SARS-CoV-2 M^*pro*^

**FIGURE 5:**
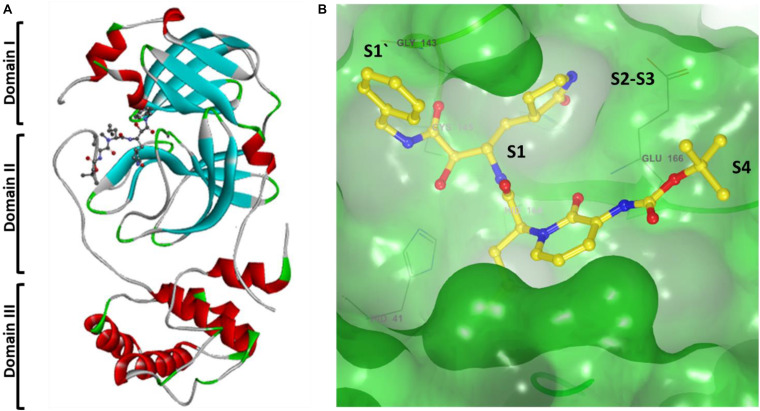
**(A)** Cartoon representation of one protomer of the dimeric M^*pro*^–inhibitor complex. **(B)** Surface presentation of conserved substrate-binding pockets of M^*pro*^ from coronaviruses. The key residues that form the binding pocket are shown in sticks, conserved substitution in M^*pro*^ of more than one of the coronaviruses. S1, S2, S4, and S1′ subsites are indicated.

Recently SARS-CoV-2 M^*pro*^ X-ray crystal structure elucidated, which showed with an α-ketoamide as a potent inhibitor in the enzyme’s active site, and screened for several FDA approved antiviral drugs and thereby blocking the active pocket (Kumar et al., 2020; Zhang N. et al., 2020). The structure of SARS-CoV-2 M^*pro*^ in the apo form and α-ketoamide bound form shows that the protein makes a crystallographic dimer composed of two monomers of identical conformations. Each protomer is furthermore made up of three domains. The interface of domain I and domain II form the active site of the protein, which is composed of a Cys145-His41 dyad where α -ketoamide derivative bound (Figures 5A,B). Domain I (residues 8–101) and domain II (residues 102–184) have an antiparallel β-barrel structure. Domain III (residues 201–303) contains five α-helices arranged into a largely antiparallel globular cluster, and it is connected to domain II by a long loop region (residues 185–200). SARS-CoV-2 M^*pro*^ has a Cys-His catalytic dyad, and the substrate-binding site is located in a cleft between domain I and domain II. These features are similar to previously reported M^*pro*^ from other coronaviruses (Anand, 2002; Yang et al., 2003; Xue et al., 2008; Wang et al., 2016; Ren et al., 2020). It is stabilized by several interactions with the active site residues His41 and Cys145 and adjacent residues in substrate binding cleft such as Gly143 and Ser144. Coronaviruses use a chymotrypsin-like a protease (3CLpro) and a papain-like protease (PLpro) to process and cleaves its long polyprotein precursor into individually functional non-structural proteins. The active site residues are thoroughly conserved and make a catalytic Cys145-His41 dyad. The specific subsite residues located in the enzyme active site are named as S1‘, S1, S2, S3, and S4 depending on their relative position to the cleavage site and subsites P1′, P1, P2, P3, and P4 in the polyprotein. Subsite P1 corresponds to the amino acid just before the cleavage site, and position P1 corresponds to the residue immediately after the cleavage site (Anand, 2003; Kiemer et al., 2004). In the M^*pro*^ of SARS-CoV-2 active site region, the S1′ residues are contributed by Cys145, Gly143, and Ser144 which also serve as the oxyanion hole. The S1 residue is His163, while Glu166 and Gln189 located at the S2 position. Bulky Gln189 and Pro168 make the S4 site (Figure 5A). The main protease recognizes and binds specific residues at each subsite of the peptide substrate to determine the initiation of proteolysis and the production of non-structural proteins for the formation of the replication-transcription complex.

A previous study proposed that M^*pro*^ has a substrate-recognition pocket that is highly conserved among all coronaviruses and that this pocket could serve as a drug target for the design of broad-spectrum inhibitors. The recent discovery of new coronaviruses, and the accumulation of structural data for M^*pro*^ from coronaviruses of various species, provided the opportunity to further examine this hypothesis. Crystal structures of M^*pro*^ revealed that (Xue et al., 2008; Wang et al., 2016; Ren et al., 2020) most variable regions are the helical domain III and surface loops, and that the substrate-binding pocket (located in a cleft between domain I and domain II) is highly conserved among M^*pro*^ in all coronaviruses; this suggests that antiviral inhibitors targeting this pocket should have wide-spectrum activity against coronaviruses as shown in Figure 5B.

## Development of MERS-CoV Vaccines

SARS-CoV-2 has caused a global public health crisis, and a vaccine urgently needs to be developed. An effective vaccine will also help control the pandemic situation of the emerging SARS virus and act as an effective treatment against its genotypes and stereotypes (Figure 2; Pawelec and Weng, 2020). Researchers have also been investigating live-attenuated vaccines against SARS in animal models, which may be helpful for SARS-CoV-2 (Aurélien et al., 2020). There are three possible targets for the development of a SARS-CoV-2 vaccine. Everyone has different types of immunity level l, particularly during old age and in children or those suffering a non-communicable disease, and it is therefore highly difficult to optimize a single vaccine for all age groups. RNA-based SARS-CoV-2 vaccines are also in development (Table 4). Adjuvants may help enhance the immune response when such vaccines are administered to older populations and may reduce the amount of RNA needed in each vaccine (Hotez et al., 2020). After the Wuhan outbreak, Chinese scientists published the genome sequence of SARS-CoV-2 and started designing a vaccine. Traditional methods required an actual sample of the virus. When an inactivated virus is injected into the body, the immune system is recognized as an antigen. Vaccines can be used as antigens in the body to protect against SARS-CoV-2 (Ojha et al., 2020). For vaccine development, a rapid response after direct injection of antigen into the body is required. These types of vaccines typically send instructions into the cell of the body. Because the cells produce an antigen protein specific to the virus a suitable design is needed. These instructions are in the form of RNA or DNA 19 (Zhang N. et al., 2020). The molecules contain the code for different proteins. This process requires a shorter development time because we do not need to grow the entire virus. Scientists have tried to replicate a SARS-CoV-2-like sequence designed like a cassette tray and slot for use in new virus antigens. Scientists have tried to like COVID-19 that sequence designed like cassette trey and slut in new virus antigens. To deal with SARS-CoV-2, CEPI is funding several teams globally, each independently working from a plate form model. As a nucleic acid-based candidate, NIH (United States) is developing a vaccine on an mRNA platform. They applied a conventional approach using the body’s cells rather than a lab test producing antigenic proteins, that’s been once made by like SARS-CoV-2. The host immune system then stimulates and develops antibodies to SARS-CoV-2. It can immediately recognize SARS-CoV-2 later and prevent the person from becoming sick. Scientists are also currently working on preventing sickness from SARS-CoV-2 (Wang F. et al., 2020). As the race for a novel and viable vaccine continues for all age groups, we do not know how bad the situation will become. Hopefully, a vaccine will soon be ready for distribution. Normally, a vaccine takes years to reach the clinical stage. However, researchers are working hard using high throughput analyses to identify the SARS-CoV-2 sequence. A small genetic sequence of the virus, received globally, is applied, and a computer algorithm scans the sequence to find tiny motifs in the DNA. A strong immune response is needed to fight against SARS-CoV-2. The moment the genetic code for the 2019 novel coronavirus was released by the Chinese government, the team at INOVIO Pharmaceuticals in California, United States began developing a new DNA medicine to kill it (Arnold, 2020). They identified, such motifs, inserted another piece of plasmid, and amplified it using bacteria to create new DNA. They then purified the DNA and injected it into completely healthy cells of a human body. This DNA acts as a map or picture of the human body. It finds the SARS-CoV-2 and attacks and kills it. There is nothing on the market yet for DNA medicines. This will be ready for human clinical trials by the end of June 2020. Since 2016, Migal Galilee Research Institute, an Israeli company, has been working on a poultry coronavirus. According to an expert in this field, poultry coronaviruses are similar to human coronaviruses, and they are more likely to produce a SARS-CoV-2 vaccine in a short period (Hodgson, 2020). Researchers are optimistic about overcoming the major challenges and safety issues. Recent developments in vaccines against SARS-CoV-2 are shown in Table 4.

**TABLE 4:** Tabular representation of ongoing clinical studies of vaccines for SARS-CoV-2.

**Vaccine candidate**	**Details**	**Status**	**Organizations**	**References**
Bacillus Calmette-Guerin (BCG) live-attenuated vaccine for SARS-CoV-2	BCG vaccine may be effective in preventing acute respiratory tract infections in elderly patients, other respiratory infection and sepsis. in the fight against SARS-CoV-2	Phase 3 BRACE trial in Australia, Netherlands	University of Melbourne and Murdoch Children’s Research Institute (Australia); Radboud University Medical Center (Netherlands); Faustman Lab at Massachusetts General Hospital (MGH) (United States)	Kamat and Kumari, 2020
mRNA-1273	mRNA-1273, a vaccine candidate based on previous study of SARS and MERS	Phase 2 clinical trial	Kaiser Permanente Washington Health Research Institute	Jackson et al., 2020
Ad5-nCoV	Recombinant novel corona virus vaccine with adenovirus type 5 vector (Ad5)	Phase 2 trial	Tongji Hospital, Wuhan, China	Feng L. et al., 2020
ChAdOx1	SARS-CoV-2, adenovirus vaccine vector MERS vaccine.	Phase 1/2 clinical trial	The University of Oxford	van Doremalen et al., 2020
INO-4800	DNA vaccine for SARS-CoV-2	Phase 1 trial	Inovio Pharmaceuticals	Smith et al., 2020
BNT162	Modified mRNA-based, SARS-CoV-2 vaccine	Phase 1/2 trial	Pfizer and BioNTech	Keech et al., 2020
NVX-CoV2373	Recombinant nanoparticle vaccine candidates for SARS-CoV-2	Phase 1 trial	Novavax, United States	Thanh et al., 2020
CureVac	mRNA-based SARS-CoV-2 vaccine	Under progress	CureVac AG, a clinical stage biopharmaceutical	Wu R. et al., 2020
Vaxart	Oral recombinant SARS-CoV-2 vaccine; gene-based vaccine	Phase 1 clinica	Emergent Biosolutions	Maurya et al., 2020
DNA vaccine candidates	DNA-based vaccine for SARS-CoV-2	Preclinical	Takis Biotech, Italy	Frederiksen et al., 2020
mRNA vaccine	Repurposed SARS vaccine and mRNA vaccine candidate	Lab testing	Sanofi, Paris, France	Ng et al., 2020
DNA plasmid vaccine candidate	Modified vaccinia ankara virus like particles (MVA-VLP) vaccine candidate for SARS-CoV-2	Pre-clinical testing	GeoVax and BravoVax, China	Chen W. H. et al., 2020
Adenovirus-based vector vaccine for SARS-CoV-2	Adenovirus-based vector vaccine for SARS-CoV-2	Animal testing	Greffex Inc., Aurora, CO, United States	Funk et al., 2020
Modified avian coronavirus vaccine	Genetically similar avian coronavirus Infectious Bronchitis Virus	Ready for human trials	*MIGAL – Galilee Research Institute, Israel*	Zhu et al., 2020
Gene-encoded antibody vaccine candidate	Next-generation, gene-encoded antibody vaccine for SARS-CoV-2	Phase I	Sorrento Therapeutics, Inc., and SmartPharm Therapeutics Inc., United States	Folegatti et al., 2020
DPX- SARS-CoV-2	T-cell activating immunotherapy antigen vaccine	Phase 1 clinical	IMV, Canada	Rabaan et al., 2020
Intranasal DNA-based vaccine candidate	Stimulating an immune response in the nasal cavity	Phase 1 clinical	University of Waterloo Waterloo, ON, Canada	Folegatti et al., 2020
Single-dose patch delivery vaccine	Vaccine candidate for SARS-CoV-2 delivered through a single-dose patch	Testing in Animal	California-based Biotech company Verndari, United States	Sharpe et al., 2020

## Epidemiology

Most of the Wuhan cases had links with the wet seafood market. The etiology was studied in a patient who was admitted to a health center and similar types of clinical signs and symptoms were found (Gangqiang et al., 2020). Approximately 40 days later, it was determined that the virus belongs to group 2 coronaviruses of bats and was 70% like the genome of SARS-CoV (Chen W. H. et al., 2020). SARS-CoV-2 is closely related to bat coronaviruses, pangolin coronaviruses, and SARS-CoV. The genome and genetic shift of such coronaviruses are shown in Figure 2. A similar type of outbreak occurred during the middle of January of 2003 until the last week of February of the same year, during the Spring Festival, or Lunar New Year, in China (Ali et al., 2020). This newest outbreak also peaked between the first and third weeks of January 2020 (Wu P. et al., 2020). The number of travelers increased by approximately 1.7-fold during the holiday. This is also one of the reasons for the spread of SARS-CoV-2 and difficulty in identifying the disease (Jiang et al., 2020). The first person with SARS-CoV-2 symptoms was observed earlier on December 1, 2019, but was not associated with the seafood market in Wuhan. In the early stage of the cluster, two-thirds of the cases were associated with the wet market (Chan et al., 2020). On March 13, 2020, a non-authenticated report from South China Morning Post stated that a 55-year-old from Hubei was the first observed case on November 17, 2019 (Chan et al., 2020). The fatality was recorded at a rate of 2.3%. The maximum number of cases was observed on February 12, 2020; owing to modifications in the diagnosis, the classification of 13,332 (rather than laboratory) confirmed cases were all recorded as new cases on February 12, 2020, despite being diagnosed during the preceding days and weeks as per the WHO on March 11, 2020. SARS-CoV-2 is affecting more than 185 countries throughout the world. New cases in new countries are still occurring. For the distribution of cases worldwide, 80,849 (51.36%) cases were observed in mainland China, and 76,562 (48.64%) cases were recorded in the rest of the world. On March 11, 2020, the WHO declared a pandemic and the total number of cases increased globally. Positive cases are considered in individuals who have been tested positive for SARS-CoV-2 as per the standard guidelines of the WHO and CDC (Operations Dashboard for ArcGIS, 18 April 2020). During the early stage up to January 23, 2020, approximately 86% of SARS-CoV-2 infected individuals had not been detected, and these infected people were the source for approximately 79% of the later confirmed cases. The numbers of infected people are higher in many countries than the number of reported cases (Li et al., 2020). According to reports published by various countries, testing data showed that an average of 1.4% of their total populations had been infected (Li et al., 2020). Based on preliminary results, 15% tested positive for antibodies in Gageite, North Rhine-Westphalia, Germany. Mothers and pregnant women in New York City also tested positive against SARS-CoV-2, which also suggests that the number of cases presented globally was higher than previously confirmed (Streeck, 2020). A low number of cases were observed in China for those below 20 years of age, however (Sutton et al., 2020). It is not clear whether this is due to less developed symptoms or a smaller number of infections (China: Age distribution of novel coronavirus patients, 2020). In a China-based study, children were infected by adults, which needs to be validated (Bi et al., 2020). In the current stage, the basic reproduction number (Ro) of SARS-CoV-2 is 5.7, although in January 2020 it was approximately 2.5 (Ward, 2020). This may vary by population and country (Sanche et al., 2020). It is expected that by the third week of May 2020, the value will be 1.0 in many countries. Most infected individuals have recovered and the onset of symptoms and death is 6 to 14 days (Worldwide Sars-Cov-2 Statistics, 2020). Death due to SARS-CoV-2 is considered as a patient who has died after testing positive, according to the WHO and CDC guidelines (New York Times, January 23, 2020).

On January 9, 2020, the first confirmed death from SARS-CoV-2 was reported in China, and the first death outside China was reported on February 1, 2020, in the Philippines (Rothan and Byrareddy, 2020). On February 14, 2020, the first death outside Asia was reported in France. On February 5, 2020, approximately 80% of deaths in China were recorded in those above 60 years in age, with cardiovascular disease, diabetes, or in immunocompromised individuals (Johns Hopkins University SARS-CoV-2 Dataset, April 25, 2020). In some countries, the death rate is higher than normal, such as in the United States (New York City), France (Paris), and Italy. The high mortality rate may be due to strained medical facilities (Coronavirus: Is SARS-CoV-2 cause of all the fatalities in Italy, April 16, 2020). According to data available on the Johns Hopkins University website, the global death rate is 6.8%, which may vary by country. The global case fatality rate is 0.82% and the infection fatality rate is 0.1%, which may vary by country according to the University of Oxford’s Centre for Evidence-Based Medicine (Oke and Heneghan, 2020).

## Prevention

Suspected individuals should be monitored, and their health status, such as fever and difficulty in breathing, should be recorded. In case of any relevant symptoms, the individual should be isolated immediately, and nucleic acid detection for SARS-CoV-2 should be performed using an appropriate sample. Healthy individuals are advised to avoid traveling to disease outbreak areas. In general, people should stay away from crowded places and maintain more than 6 feet or 2 m from anyone with symptoms (Gangqiang et al., 2020). Wearing a surgical mask is recommended owing to the general risk of obtaining SARS-CoV-2, and hand washing is a key and should be done using soap or an alcohol-based hand sanitizer. Besides, people should avoid touching their eyes, nose, or mouth, which are known as the T-Zone and are common entry points for the viruses in the body. For healthcare workers around patients with SARS-CoV-2 (WHO, 2020; Worldwide Sars-Cov-2 Statistics, 2020), the recommendations are to avoid respiratory droplets and take precautions, which include wearing personal protective gear such as a clean and dry surgical mask and gloves, long sleeve gowns, and eye protection including goggles or a face shield (Eurosurveillance editorial team, 2020). When conducting procedures that result in aerosol-like tracheal intubation, cardiopulmonary resuscitation, or non-invasive ventilation, it is important to wear an N-95 respirator, which prevents 95% of small particles such as respiratory droplets from passing through (Güner et al., 2020).

### Prospects

Like any other disease model, the forecast of SARS-CoV-2 aims to determine when the outbreak will stop and how many people suffer or die. We need to employ novel efforts rather than generating premature investigations. Researchers have been working tirelessly since the first phase of the epidemic. At the same time, it is also necessary to develop advanced models to handle the presently available data. Scott Layne, an epidemiologist at the University of California, Los Angeles has proposed a new data bank. In addition, bioinformatician investigators are working to compile a dataset and build an algorithm. Scientists from the University of Montreal have also published chest X-ray and CT scan data for use by other researchers. Johns Hopkins University has developed a server that is regularly updated for further research on the vaccine and drug development. Researchers from Shanghai have developed a server for checking CT scan images to reduce the diagnosis (Zhang Z. et al., 2020). Researchers from the United States have claimed to have identified the effects of SARS-CoV-2 in the lungs during active infection. Wang Y. et al. (2020) has developed an auditory system-based SARS-CoV-2 detection, which is based on the breathing rate. Although this investigation has not yet been validated, the idea is sound. In addition, investigators in Wuhan, China have developed a heath calculator using nearly 3,000 electronic health records from patients, achieving more than 90% accuracy (Butt et al., 2020). Some clinicians have used anti-HIV medicine to cure SARS-CoV-2 patients, although other scientists have suggested that nanoparticle-based medicine would be more effective to fight against SARS-CoV-2 (Yan et al., 2020). Many scientists are also working on theragnostic-based approaches. To avoid research gaps in SARS-CoV-2, clinical, and public health strategies need to be implemented using more advanced tools and approaches with international cooperation. Some key points should be monitored for strategic prevention, such as a strategy for identifying contact with infected individuals, the range of virus mutation at the global level, the identification of super spreaders, the association of disease progression level of viral contagion after recovery of positive patents, immunity level between severe and non-severe cases, the immunopathological mechanism of mucous hypersecretion with a cytokine storm, effective clinical biomarkers, effective implication of artificial intelligence, identification of the optimal incubation period, and the development of cost-effective diagnosis treatment and vaccines. There is an urgent requirement to recognize and eliminate any barriers that would affect rapid patient care and public health management against the SARS-CoV-2 pandemic. Furthermore, improved effectiveness in care is urgently needed and a new approach to international coordination is required.

## Author Contributions

DDS and DKY conceived and designed the project. IH, E-HC, and DKY collected the data from the literature. DDS, IH, E-HC, and DKY analyzed the data and wrote the manuscript. All authors contributed to the interpretation and discussion of the results, and read and approved the final version of the manuscript.

## Conflict of Interest

The authors declare that the research was conducted in the absence of any commercial or financial relationships that could be construed as a potential conflict of interest.

## References

[B1] Abd El-Aziz T. M. Stockand J. D. (2020). Recent progress and challenges in drug development against SARS-COV-2 coronavirus (SARS-CoV-2) - an update on the status. *Infect. Genet Evol.* 83:104327. 10.1016/j.meegid.2020.104327 32320825 PMC7166307

[B2] Abed Y. Fage C. Checkmahomed L. Venable M.-C. Boivin G. (2020). Characterization of contemporary influenza B recombinant viruses harboring mutations of reduced susceptibility to baloxavir marboxil, in vitro and in mice. *Antiviral Res.* 179:104807. 10.1016/j.antiviral.2020.104807 32343991

[B3] Addie D. D. (2020). *Feline Coronavirus and Feline Infectious Peritonitis Diagnosis and Prevention.* Available online at: catvirus.com

[B4] Addie D. D. le Pode R S. Burr P. Decaro N. Graham E. Hofmann-Lehmann R. (2020). Utility of feline coronavirus antibody tests. *J. Feline Med. Surg.* 17 152–162. 10.1177/1098612X14538873 24966245 PMC10816424

[B5] Adnan S. (2020). Novel coronavirus-induced NLRP3 inflammasome activation: a potential drug target in the treatment of SARS-COV-2. *Front. Immunol.* 11:1021. 10.3389/fimmu.2020.01021 32574259 PMC7248552

[B6] Ali A. Areeba A. Sana H. (2020). Comparison of epidemiological variations in SARS-COV-2 Patients inside and outside of china—a meta-analysis. *Front. Public Health* 8:193. 10.3389/fpubh.2020.00193 32574293 PMC7226658

[B7] Alia E. Grant-Kels J. M. (2020). Does hydroxychloroquine combat SARS-COV-2? A timeline of evidence. *J. Am. Acad. Dermatol.* 83 e33–e34. 10.1016/j.jaad.2020.04.031 32283236 PMC7151328

[B8] Anand K. (2002). Structure of coronavirus main proteinase reveals combination of a chymotrypsin fold with an extra alpha-helical domain. *EMBO J.* 21 3213–3224. 10.1093/emboj/cdf327 12093723 PMC126080

[B9] Anand K. (2003). Coronavirus main proteinase (3CLpro) structure: basis for design of Anti-SARS Drugs. *Science* 300 1763–1767. 10.1126/science.1085658 12746549

[B10] Arnold C. (2020). Race for a vaccine. *New Sci.* 245 44–47. 10.1016/s0262-4079(20)30600-x PMC7269716 32518454

[B11] Aurélien M. Lucie H. Jean-Luc S. Jean-Philippe L. Michel B. (2020). Therapeutic options for SARS-COV-2 -modulation of type I interferon response as a promising strategy? *Cureus* 12:e10480. 10.3389/fpubh.2020.00185 32574289 PMC7243823

[B12] Azin T. Mahta A. Yeganeh F. Parnian J. Saba H. Tess M. C. (2020). Clinical features, diagnosis, and treatment of SARS-COV-2 in hospitalized patients: a systematic review of case reports and case series. *Front. Med.* 7:231. 10.3389/fmed.2020.00231 32574328 PMC7242615

[B13] Baig A. M. Khaleeq A. Ali U. Syeda H. (2020). Evidence of the SARS-COV-2 virus targeting the CNS: tissue distribution, host-virus interaction, and proposed neurotropic mechanisms. *ACS Chem. Neurosci.* 11 995–998. 10.1021/acschemneuro.0c00122 32167747

[B14] Beigel J. H. Tomashek K. M. Dodd L. E. Mehta A. K. Zingman B. S. Kalil A. C. (2020). Remdesivir for the treatment of SARS-CoV-2 - preliminary report. *New Engl. J. Med.* 2020:NEJMoa2007764. 10.1056/NEJMoa2007764 32649078

[B15] Belouzard S. Millet J. K. Licitra B. N. Whittaker G. R. (2012). Mechanisms of coronavirus cell entry mediated by the viral spike protein. *Viruses* 4 1011–1033. 10.3390/v4061011 22816037 PMC3397359

[B16] Bernard Stoecklin S. Rolland P. Silue Y. Mailles A. Campese C. Simondon A. (2020). First cases of coronavirus disease 2019 (SARS-COV-2) in France: surveillance, investigations and control measures, January 2020. *Eur. Commun. Dis. Bull.* 25:2000094. 10.2807/1560-7917.ES.2020.25.6.2000094 32070465 PMC7029452

[B17] Bi Q. Wu Y. Mei S. Ye C. Zou X. Zhang Z. (2020). Epidemiology and transmission of SARS-COV-2 in 391 cases and 1286 of their close contacts in Shenzhen, China: a retrospective cohort study. *Lancet* 202:287 10.1016/S1473-3099(20)30287-5 PMC7185944 32353347

[B18] Bleibtreu A. Jaureguiberry S. Houhou N. Boutolleau D. Guillot H. Vallois D. (2018). Clinical management of respiratory syndrome in patients hospitalized for suspected Middle East respiratory syndrome coronavirus infection in the Paris area from 2013 to 2016. *BMC Infect. Dis.* 18:331. 10.1186/s12879-018-3223-5 30012113 PMC6048819

[B19] Boulware D. R. Pullen M. F. Bangdiwala A. S. Pastick K. A. Lofgren S. M. Okafor E. C. (2020). A randomized trial of hydroxychloroquine as postexposure prophylaxis for Covid-19. *New Engl. J. Med.* 383 517–525. 10.1056/NEJMoa2016638 32492293 PMC7289276

[B20] Broughton J. P. Deng X. Yu G. Clare L. F. Venice S. Jasmeet S. (2020). CRISPR-Cas12-based detection of SARS-CoV-2. *Nat. Biotechnol.* 38 870–874. 10.1038/s41587-020-0513-4 32300245 PMC9107629

[B21] Butt C. Gill J. Chun D. Babu B. A. (2020). Deep learning system to screen coronavirus disease 2019 pneumonia. *Appl. Intellig.* 22 1–7. 10.1007/s10489-020-01714-3 PMC7175452 38624372

[B22] Cao B. Wang Y. Wen D. Liu W. Wang J. Fan G. (2020). A trial of lopinavir-ritonavir in adults hospitalized with severe SARS-CoV-2. *N. Engl. J. Med.* 382 1787–1799. 10.1056/NEJMoa2001282 32187464 PMC7121492

[B23] Cao Y. C. Deng Q. X. Dai S. X. (2020). Remdesivir for severe acute respiratory syndrome coronavirus 2 causing SARS-COV-2: an evaluation of the evidence. *Travel Med. Infect. Dis.* 35:101647. 10.1016/j.tmaid.2020.101647 32247927 PMC7151266

[B24] Chan J. F.-W. Yuan S. Kok K.-H. To K. K.-W. Chu H. Yang J. (2020). A familial cluster of pneumonia associated with the 2019 novel coronavirus indicating person-to-person transmission: a study of a family cluster. *Lancet* 395 514–523. 31986261 10.1016/S0140-6736(20)30154-9 PMC7159286

[B25] Chan J. W. M. Ng C. K. Chan Y. H. Lee S. Chu S. Law W. (2003). Short term outcome and risk factors for adverse clinical outcomes in adults with severe acute respiratory syndrome (SARS). *Thorax* 58 686–689. 12885985 10.1136/thorax.58.8.686 PMC1746764

[B26] Chan-Yeung M. Xu R. H. (2003). SARS: epidemiology. *Respirology* 8(Suppl.), S9–S14. 10.1046/j.1440-1843.2003.00518 15018127 PMC7169193

[B27] Chen N. Zhou M. Dong X. Qu J. Gong F. Yang H. (2020). Epidemiological and clinical characteristics of 99 cases of 2019 novel coronavirus pneumonia in Wuhan, China: a descriptive study. *Lancet* 395 507–513. 32007143 10.1016/S0140-6736(20)30211-7 PMC7135076

[B28] Chen W. H. Strych U. Hotez P. J. Bottazzi M. E. (2020). The SARS-CoV-2 vaccine pipeline: an overview. *Curr. Trop. Med. Rep.* 2020 1–4. 10.1007/s40475-020-00201-6 32219057 PMC7094941

[B29] Chen X. Yu B. (2020). First two months of the 2019 Coronavirus disease (SARS-COV-2) epidemic in China: real-time surveillance and evaluation with a second derivative model. *Glob. Health Res. Policy* 5:7. 10.1186/s41256-020-00137-4 32158961 PMC7050133

[B30] Coenen S. van der Velden A. W. Cianci D. Goossens H. Bongard E. Saville B. R. (2020). Oseltamivir for coronavirus illness: post-hoc exploratory analysis of an open-label, pragmatic, randomised controlled trial in European primary care from 2016 to 2018. *Br. J. Gen. Pract.* 70 e444–e449. 10.3399/bjgp20X711941 32571773 PMC7311109

[B31] Coleman C. M. Frieman M. B. (2015). Growth and quantification of MERS-CoV infection. *Curr. Protoc. Microbiol.* 37 11–15. 10.1002/9780471729259.mc15e02s37 26344219 PMC4735735

[B32] Corman V. M. Landt O. Kaiser M. Molenkamp R. Meijer A. Chu D. (2020). Detection of 2019 novel coronavirus (2019-nCoV) by real-time RT-PCR. *Eur. Commun. Dis. Bull.* 25:2000045. 10.2807/1560-7917.ES.2020.25.3.2000045 31992387 PMC6988269

[B33] Cowling B. J. Leung G. M. (2020). Epidemiological research priorities for public health control of the ongoing global novel coronavirus (2019-nCoV) outbreak. *Eur. Commun. Dis. Bull.* 25:2000110. 10.2807/1560-7917.ES.2020.25.6.2000110 32046814 PMC7029449

[B34] Cui P. Chen Z. Wang T. Dai J. Zhang J. Ding T. (2020). Severe acute respiratory syndrome coronavirus 2 detection in the female lower genital tract. *Am. J. Obstetr. Gynecol.* 223 131–134. 10.1016/j.ajog.2020.04.038 32376320 PMC7196539

[B35] Du Y. Tu L. Zhu P. Mu M. Wang R. Yang P. (2020). Clinical features of 85 Fatal cases of SARS-COV-2 from Wuhan. A retrospective observational study. *Am. J. Respirat. Crit. Care Med.* 201 1372–1379. 10.1164/rccm.202003-0543OC 32242738 PMC7258652

[B36] Eastman R. T. Roth J. S. Brimacombe K. R. Simeonov A. Shen M. Patnaik S. (2020). Remdesivir: a review of its discovery and development leading to emergency use authorization for treatment of SARS-COV-2. *ACS Cent. Sci.* 6 672–683. 10.1021/acscentsci.0c00489 32483554 PMC7202249

[B37] Elena C. Carmine V. Annibale A. P. (2020). SARS-COV-2 infection and circulating ACE2 levels: protective role in women and children. *Front. Pediatr.* 8:206. 10.3389/fped.2020.00206 32391299 PMC7192005

[B38] Eurosurveillance editorial team (2020). Note from the editors: novel coronavirus (2019-nCoV). *Eurosurveillance* 25:2001231. 10.2807/1560-7917.Es.2020.25.3.2001231 31992390 PMC6988271

[B39] Feng L. Wang Q. Shan C. Yang C. Feng Y. Wu J. (2020). An adenovirus-vectored COVID-19 vaccine confers protection from SARS-COV-2 challenge in rhesus macaques. *Nat. Commun.* 11:4207. 10.1038/s41467-020-18077-5 32826924 PMC7442803

[B40] Feng Y. Ling Y. Bai T. Xie Y. Huang J. Li J. (2020). SARS-COV-2 with different severities: a multicenter study of clinical features. *Am. J. Respir. Crit. Care Med.* 201 1380–1388. 10.1164/rccm.202002-0445OC 32275452 PMC7258639

[B41] Ferner R. E. Aronson J. K. (2020). Chloroquine and hydroxychloroquine in SARS-CoV-2. *BMJ* 369:m1432. 10.1136/bmj.m1432 32269046

[B42] Folegatti P. M. Ewer K. J. Aley P. K. Angus B. Becker S. Belij-Rammerstorfer S. (2020). Safety and immunogenicity of the ChAdOx1 nCoV-19 vaccine against SARS-CoV-2: a preliminary report of a phase 1/2, single-blind, randomised controlled trial. *Lancet* 396 467–478. 10.1016/s0140-6736(20)31604-4 32702298 PMC7445431

[B43] Frederiksen L. S. F. Zhang Y. Foged C. Thakur A. (2020). The long road toward COVID-19 herd immunity: vaccine platform technologies and mass immunization strategies. *Front. Immunol.* 11:1817. 10.3389/fimmu.2020.01817 32793245 PMC7385234

[B44] Funk C. D. Laferrière C. Ardakani A. (2020). A snapshot of the global race for vaccines targeting SARS-CoV-2 and the COVID-19 pandemic. *Front. Pharmacol.* 11:937. 10.3389/fphar.2020.00937 32636754 PMC7317023

[B45] Gangqiang G. Lele Y. Kan P. Yu C. Dong X. Kejing Y. (2020). New insights of emerging SARS-CoV-2:epidemiology, etiology, clinical features, clinical treatment and prevention. *Front. Cell Dev. Biol.* 8:410. 10.3389/fcell.2020.00410 32574318 PMC7256189

[B46] Ge X. Y. Li J. L. Yang X. L. Chmura A. A. Zhu G. Epstein J. H. (2013). Isolation and characterization of a bat SARS-like coronavirus that uses the ACE2 receptor. *Nature* 503 535–538. 10.1038/nature12711 24172901 PMC5389864

[B47] Geleris J. Sun Y. Platt J. Zucker J. Baldwin M. Hripcsak G. (2020). Observational study of hydroxychloroquine in hospitalized patients with Covid-19. *New Engl. J. Med.* 382 2411–2418. 10.1056/NEJMoa2012410 32379955 PMC7224609

[B48] Giuseppe D. L. Rossella D. T. (2020). Coronavirus disease (SARS-COV-2) in Italy: analysis of risk factors and proposed remedial measures. *Front. Med.* 7:140. 10.3389/fmed.2020.00140 32328496 PMC7161343

[B49] Goossens G. A. (2015). Flushing and locking of venous catheters: available evidence and evidence deficit. *Nurs. Res. Pract.* 2015:985686. 10.1155/2015/985686 26075094 PMC4446496

[B50] Gordon C. J. Tchesnokov E. P. Feng J. Y. Porter D. P. Götte M. (2020). The antiviral compound remdesivir potently inhibits RNA-dependent RNA polymerase from Middle East respiratory syndrome coronavirus. *J. Biol. Chem.* 295 4773–4779. 10.1074/jbc.AC120.013056 32094225 PMC7152756

[B51] Güner R. Hasanoğlu I. Aktaş F. (2020). SARS-COV-2: prevention and control measures in community. *Turk. J. Med. Sci.* 50 571–577. 10.3906/sag-2004-146 32293835 PMC7195988

[B52] Hodgson J. (2020). The pandemic pipeline. *Nat. Biotechnol.* 38 523–532. 10.1038/d41587-020-00005-z 32203293

[B53] Hong K. H. Lee S. W. Kim T. S. Hee J. Lee J. Kim S. Y. (2020). Guidelines for laboratory diagnosis of coronavirus disease 2019 (SARS-COV-2) in Korea. *Ann. Lab. Med.* 40 351–360. 10.3343/alm.2020.40.5.351 32237288 PMC7169629

[B54] Horby P. (2020). Dexamethasone in hospitalized patients with Covid-19-preliminary report. *New Engl. J. Med.* 10.1056/NEJMoa2021436 32678530 PMC7383595

[B55] Hotez P. J. Corry D. B. Bottazzi M. E. (2020). SARS-COV-2 vaccine design: the Janus face of immune enhancement. *Nat. Rev. Immunol.* 20:347. 10.1038/s41577-020-0323-4 PMC7187801 32346094

[B56] Huang C. Wang Y. Li X. Ren L. Zhao J. Hu Y. (2020). Clinical features of patients infected with 2019 novel coronavirus in Wuhan, China. *Lancet* 395 497–506. 31986264 10.1016/S0140-6736(20)30183-5 PMC7159299

[B57] Huang D. Yu H. Wang T. Yang H. Yao R. Liang Z. (2020). Efficacy and safety of umifenovir for coronavirus disease 2019 (COVID-19): a systematic review and meta-analysis. *J. Med. Virol.* 2020:jmv.26256. 10.1002/jmv.26256 32617989 PMC7361300

[B58] Jackson L. A. Anderson E. J. Rouphael N. G. Roberts P. C. Makhene M. Coler R. N. (2020). An mRNA vaccine against SARS-CoV-2-preliminary report. *New Engl. J. Med.* 10.1056/NEJMoa2022483 32663912 PMC7377258

[B59] Jeong G. U. Song H. Yoon G. Y. Kim D. Kwon Y.-C. (2020). Therapeutic strategies against COVID-19 and structural characterization of SARS-CoV-2: a review. *Front. Microbiol.* 11:1723. 10.3389/fmicb.2020.01723 32765482 PMC7381222

[B60] Jiang F. Deng L. Zhang L. Cai Y. Cheung C. W. Xia Z. (2020). Review of the clinical characteristics of Coronavirus disease 2019 (SARS-COV-2). *J. Gen. Intern. Med.* 35 1545–1549. 10.1007/s11606-020-05762-w 32133578 PMC7088708

[B61] Jian-Min J. Peng B. Wei H. Fei W. Li X. F. Shi L. (2020). Gender differences in patients with SARS-COV-2: focus on severity and mortality. *Front. Public Health* 8:152. 10.3389/fpubh.2020.00152 32411652 PMC7201103

[B62] Kamat S. Kumari M. (2020). BCG against SARS-CoV-2: second youth of an old age vaccine? *Front. Pharmacol.* 11:1050. 10.3389/fphar.2020.01050 32754036 PMC7381314

[B63] Keech C. Albert G. Cho I. Robertson A. Reed P. Neal S. (2020). Phase 1-2 trial of a SARS-CoV-2 recombinant spike protein nanoparticle vaccine. *New Engl. J. Med.* 21:44. 10.1056/NEJMoa2026920 32877576 PMC7494251

[B64] Khan G. (2013). A novel coronavirus capable of lethal human infections: an emerging picture. *Virol. J.* 10:66. 10.1186/1743-422X-10-66 23445530 PMC3599982

[B65] Kiemer L. Lund O. Brunak S. Blom N. (2004). Blom Coronavirus 3CLproproteinase cleavage sites: possible relevance to SARS virus pathology. *BMC Bioinform.* 5:72. 10.1186/1471-2105-5-72 15180906 PMC442122

[B66] Kruse R. L. (2020). Therapeutic strategies in an outbreak scenario to treat the novel coronavirus originating in Wuhan, China. *F1000Research* 9:72. 10.12688/f1000research.22211.2 32117569 PMC7029759

[B67] Kumar Y. Singh H. Patel C. N. (2020). In silico prediction of potential inhibitors for the main protease of SARS-CoV-2 using molecular docking and dynamics simulation based drug-repurposing. *J. Infect. Public Health* 13 1210–1223. 10.1016/j.jiph.2020.06.016 32561274 PMC7297718

[B68] Lau S. K. Li K. S. Huang Y. Shek C. T. Tse H. Wang M. (2010). Ecoepidemiology and complete genome comparison of different strains of severe acute respiratory syndrome-related Rhinolophus bat coronavirus in China reveal bats as a reservoir for acute, self-limiting infection that allows recombination events. *J. Virol.* 84 2808–2819. 10.1128/JVI.02219-09 20071579 PMC2826035

[B69] Lau S. K. Li K. S. Tsang A. K. Lam C. S. Ahmed S. Chen H. (2013). Genetic characterization of Betacoronavirus lineage C viruses in bats reveals marked sequence divergence in the spike protein of pipistrellus bat Coronavirus HKU5 in Japanese pipistrelle: implications for the origin of the novel Middle East respiratory syndrome Coronavirus. *J. Virol.* 87 8638–8650. 10.1128/JVI.01055-13 23720729 PMC3719811

[B70] Letko M. Marzi A. Munster V. (2020). Functional assessment of cell entry and receptor usage for SARS-CoV-2 and another lineage B Betacoronaviruses. *Nat. Microbiol.* 5 562–569. 10.1038/s41564-020-0688-y 32094589 PMC7095430

[B71] Li K. Wu J. Wu F. Guo D. Chen L. Fang Z. (2020). The clinical and chest CT features associated with severe and critical SARS-COV-2 pneumonia. *Invest. Radiol.* 55 327–331. 10.1097/RLI.0000000000000672 32118615 PMC7147273

[B72] Liu B. Li M. Zhou Z. Guan X. Xiang Y. (2020). Can we use interleukin-6 (IL-6) blockade for coronavirus disease 2019 (SARS-COV-2)-induced cytokine release syndrome (CRS)? *J. Autoimmun.* 111:102452. 10.1016/j.jaut.2020.102452 32291137 PMC7151347

[B73] Liu K. Chen Y. Lin R. Han K. (2020). Clinical features of SARS-COV-2 in elderly patients: a comparison with young and middle-aged patients. *J. Infect.* 80 e14–e18. 10.1016/j.jinf.2020.03.005 32171866 PMC7102640

[B74] Liu H. Liu F. Li J. Zhang T. Wang D. Lan W. (2020). Clinical and CT imaging features of the SARS-COV-2 pneumonia: Focus on pregnant women and children. *J. Infect.* 80 e7–e13. 10.1016/j.jinf.2020.03.007 32171865 PMC7156118

[B75] Matthay M. A. Zemans R. L. Zimmerman G. A. Arabi Y. M. Beitler J. R. Mercat A. (2019). Acute respiratory distress syndrome. *Nat. Rev. Dis. Prim.* 5:572. 10.1038/s41572-019-0069-0 30872586 PMC6709677

[B76] Maurya C. K. Misra R. Sharma P. Singh N. Awasthi H. Agrawal R. (2020). Novel stem cells and nucleic acid-based vaccine trials against viral outbreak: a systematic evaluation during COVID-2019 pandemic. *Indian J. Clin. Biochem.* 35 397–409. 10.1007/s12291-020-00907-4 32837030 PMC7347658

[B77] McBride R. van Zyl M. Fielding B. C. (2014). The coronavirus nucleocapsid is a multifunctional protein. *Viruses* 6 2991–3018. 10.3390/v6082991 25105276 PMC4147684

[B78] Meyerowitz E. A. Vannier A. Friesen M. Schoenfeld S. Gelfand J. A. Callahan M. V. (2020). Rethinking the role of hydroxychloroquine in the treatment of SARS-COV-2. *FASEB J.* 34 6027–6037. 10.1096/fj.202000919 32350928 PMC7267640

[B79] Narayanan K. Ramirez S. I. Lokugamage K. G. Makino S. (2015). Coronavirus nonstructural protein 1: common and distinct functions in the regulation of host and viral gene expression. *Virus Res.* 202 89–100. 10.1016/j.virusres.2014.11.019 25432065 PMC4444399

[B80] Ng W. H. Liu X. Mahalingam S. (2020). Development of vaccines for SARS-CoV-2. *F1000Research* 9:25998 10.12688/f1000research.25998 PMC7431966 32850116

[B81] Ojha R. Gupta N. Naik B. Singh S. Verma V. K. Prusty D. (2020). High throughput and comprehensive approach to develop multiepitope vaccine against minacious SARS-COV-2. *Eur. J. Pharmaceut. Sci.* 151:105375. 10.1016/j.ejps.2020.105375 32417398 PMC7224663

[B82] Oke J. Heneghan C. (2020). *Global Covid-19 Case Fatality Rates; Centre for Evidence-Based Medicine.* Oxford: Oxford University.

[B83] Owusu M. Annan A. Corman V. M. Larbi R. Anti P. Drexler J. F. (2014). Human coronaviruses associated with upper respiratory tract infections in three rural areas of Ghana. *PLoS One* 9:e99782. 10.1371/journal.pone.0099782 25080241 PMC4117488

[B84] Pawelec G. Weng N. P. (2020). Can an effective SARS-CoV-2 vaccine be developed for the older population? *Immun. Age* 17:8. 10.1186/s12979-020-00180-2 32300370 PMC7148425

[B85] Puja M. McAuley D. F. Brown M. Sanchez E. Tattersall R. S. Manson J. J. (2020). COVID-19: consider cytokine storm syndromes and immunosuppression. *Cell Stress* 395 1033–1034. 10.1016/S0140-6736(20)30628-0 PMC7270045 32192578

[B86] Rabaan A. A. Al-Ahmed S. H. Sah R. Tiwari R. Yatoo M. I. Patel S. K. (2020). SARS-CoV-2/COVID-19 and advances in developing potential therapeutics and vaccines to counter this emerging pandemic. *Ann. Clin. Microbiol. Antimicrob.* 19:38 10.1186/s12941-020-0038 PMC7464065 32878641

[B87] Raoult D. Zumla A. Locatelli F. Ippolito G. Kroemer G. (2020). Coronavirus infections: epidemiological, clinical and immunological features and hypotheses. *Cell Stress* 4 66–75. 10.15698/cst2020.04.216 32292881 PMC7064018

[B88] Raza S. S. Khan M. A. (2020). Mesenchymal stem cells: a new front emerge in COVID19 treatment. *Cytotherapy* 10.1016/j.jcyt.2020.07.002 [Epub ahead of print]. PMC7362822 35880307

[B89] Ren Z. Yan L. Zhang N. Guo Y. Yang C. Lou Z. (2020). The newly emerged SARS-Like coronavirus HCoV-EMC also has an “Achilles’ heel”: current effective inhibitor targeting a 3C-like protease. *Protein Cell* 4 248–250. 10.1007/s13238-013-2841-3 23549610 PMC4875521

[B90] Rothan H. A. Byrareddy S. N. (2020). The epidemiology and pathogenesis of coronavirus disease (SARS-COV-2) outbreak. *J. Autoimmun.* 109:102433. 10.1016/j.jaut.2020.102433 32113704 PMC7127067

[B91] Russell C. D. Millar J. E. Baillie J. K. (2020). Clinical evidence does not support corticosteroid treatment for 2019-nCoV lung injury. *Lancet* 395 473–475. 32043983 10.1016/S0140-6736(20)30317-2 PMC7134694

[B92] Sae-im J. (2020). Bukwang to test its antiviral drug against Covid-19. *Korea Biomedical Rev.* Available online at: http://www.koreabiomed.com/news/articleView.html?idxno=8010

[B93] Salazar E. Perez K. K. Ashraf M. Chen J. Castillo B. Christensen P. A. (2020). Treatment of SARS-COV-2 patients with convalescent plasma. *Am. J. Pathol.* 323 1582–1589. 10.1016/j.ajpath.2020.05.014 32473109 PMC7251400

[B94] Sanche S. Lin Y. T. Xu C. Romero-Severson E. Hengartner N. Ke R. (2020). High contagiousness and rapid spread of severe acute respiratory syndrome Coronavirus 2. *Emerg. Infect. Dis.* 26:282. 10.3201/eid2607.200282 32255761 PMC7323562

[B95] Schoeman D. Fielding B. C. (2019). Coronavirus envelope protein: current knowledge. *Virol. J.* 16:69. 10.1186/s12985-019-1182-0 31133031 PMC6537279

[B96] Senanayake S. L. (2020). Drug repurposingstrategies for COVID-19. *Future Drug Discov.* 2:fdd-2020–0010. 10.4155/fdd-2020-0010

[B97] Sharpe H. R. Gilbride C. Allen E. Belij-Rammerstorfer S. Bissett C. Ewer K. (2020). The early landscape of coronavirus disease 2019 vaccine development in the UK and rest of the world. *Immunology* 160 223–232. 10.1111/imm.13222 32460358 PMC7283842

[B98] Shen L. Niu J. Wang C. Huang B. Wang W. Zhu N. (2019). High-throughput screening and identification of potent broad-spectrum inhibitors of Coronaviruses. *J. Virol.* 93:e0023-19. 10.1128/JVI.00023-19 30918074 PMC6613765

[B99] Shih-Hwa C. Pei Ching C. Yanwen L. Mong-Lien W. Chian-Hsu C. Yi-Ping Y. (2020). Highlight of immune pathogenic response and hematopathologic effect in SARS-CoV, MERS-CoV and SARS-Cov-2 infection. *Front. Immunol.* 11:1022. 10.3389/fimmu.2020.01022 32574260 PMC7236801

[B100] Singh D. D. Hawkins R. D. Lahesmaa R. Tripathi S. K. (2019). CRISPR/Cas9 guided genome and epigenome engineering and its therapeutic applications in immune mediated diseases. *Semin. Cell Dev. Biol.* 96 32–43. 31112800 10.1016/j.semcdb.2019.05.007

[B101] Singhal T. (2020). A review of Coronavirus Disease-2019 (SARS-COV-2). *Indian J. Pediat.* 87 281–286. 10.1007/s12098-020-03263-6 32166607 PMC7090728

[B102] Smith R. D. (2006). Responding to global infectious disease outbreaks: lessons from SARS on the role of risk perception, communication and management. *Soc. Sci. Med.* 63 3113–3123. 10.1016/j.socscimed.2006.08.004 16978751 PMC7130909

[B103] Smith T. R. F. Patel A. Ramos S. Elwood D. Zhu X. Yan J. (2020). Immunogenicity of a DNA vaccine candidate for COVID-19. *Nat. Commun.* 11:2601. 10.1038/s41467-020-16505-0 32433465 PMC7239918

[B104] Sodhi M. Etminan M. (2020). Safety of ibuprofen in patients with SARS-COV-2: causal or confounded? *Chest* 158 55–56. 10.1016/j.chest.2020.03.040 32243944 PMC7151542

[B105] Streeck H. (2020). *Vorläufiges Ergebnis und Schlussfolgerungen der SARS-COV-2 Case-Cluster-Study (Gemeinde Gangelt).* Available online at: https://www.land.nrw/sites/default/files/asset/document/zwischenergebnis_ covid19_case_study_gangelt_0.pdf?fbclid=IwAR2Ul47uWQOgf5SK6n1Cewm u4UKRSN7JdAIYhBX9irdAB6ZZy8klHYKdv80 (accessed April 13, 2020).

[B106] Sutton D. Fuchs K. D’Alton M. Goffman D. (2020). Universal screening for SARS-CoV-2 in Women admitted for delivery. *New Engl. J. Med.* 382 2163–2164. 10.1056/NEJMc2009316 32283004 PMC7175422

[B107] Tan K. S. Lim R. L. Liu J. Ong H. H. Tan V. J. Lim H. F. (2020). Respiratory viral infections in exacerbation of chronic airway inflammatory diseases: novel mechanisms and insights from the upper airway epithelium. *Front. Cell Dev. Biol.* 8:99. 10.3389/fcell.2020.00099 32161756 PMC7052386

[B108] Tan L. Wang Q. Zhang D. Ding J. Huang Q. Tang Y. Q. (2020). Lymphopenia predicts disease severity of SARS-COV-2: a descriptive and predictive study. *Signal Transd. Target. Ther.* 5:33. 10.1038/s41392-020-0148-4 32296069 PMC7100419

[B109] Thanh L. T. Andreadakis Z. Kumar A. Gómez Román R. Tollefsen S. Saville M. (2020). The COVID-19 vaccine development landscape. *Nat. Rev. Drug Discov.* 19 305–306. 10.1038/d41573-020-00073-5 32273591

[B110] Tian S. Hu W. Niu L. Liu H. Xu H. Xiao S. Y. (2020). Pulmonary pathology of early-phase 2019 novel Coronavirus (SARS-COV-2) pneumonia in two patients with lung cancer. *J. Thorac. Oncol.* 15 700–704. 10.1016/j.jtho.2020.02.010 32114094 PMC7128866

[B111] Udugama B. Kadhiresan P. Kozlowski H. N. Malekjahani A. Osborne M. Li V. (2020). Diagnosing SARS-COV-2: the disease and tools for detection. *ACS Nano* 14 3822–3835. 10.1021/acsnano.0c02624 32223179

[B112] van Doremalen N. Lambe T. Spencer A. Belij-Rammerstorfer S. Purushotham J. N. Port J. R. (2020). ChAdOx1 nCoV-19 vaccine prevents SARS-CoV-2 pneumonia in rhesus macaques. *bioRxiv* [Preprint]. 10.1038/s41586-020-2608-y 33469217

[B113] Verma R. Sahu R. Singh D. D. Egbo T. E. (2019). A CRISPR/Cas9 based polymeric nanoparticles to treat/inhibit microbial infections. *Semin. Cell Dev. Biol.* 96 44–52. 30986568 10.1016/j.semcdb.2019.04.007

[B114] Wang F. Chen C. Tan W. Yang K. Yang H. N. (2016). Structure of main protease from human Coronavirus NL63: insights for wide spectrum anti-Coronavirus drug design. *Sci. Rep.* 6:677. 10.1038/srep22677 26948040 PMC4780191

[B115] Wang F. Kream R. M. Stefano G. B. (2020). An evidence based perspective on mRNA-SARS-CoV-2 vaccine development. *Med. Sci. Monit.* 26:e924700. 10.12659/MSM.924700 32366816 PMC7218962

[B116] Wang Y. Wang Y. Chen Y. Qin Q. (2020). Unique epidemiological and clinical features of the emerging 2019 novel coronavirus pneumonia (SARS-COV-2) implicate special control measures. *J. Med. Virol.* 92 568–576. 10.1002/jmv.25748 32134116 PMC7228347

[B117] Ward D. (2020). *Sampling Bias: Explaining Variations in Age Distributions of SARS-COV-2 Cases.* Bern: WardEnvironment.

[B118] WHO (2020). *Ward Environment WHO Director-General’s Opening Remarks at the Media Briefing on SARS-COV-2 - 11 March 2020.* Technical Report.

[B119] Worldwide Sars-Cov-2 Statistics (2020). Available online at: https://ww.who.int/emergencies/diseases/novel-coronavirus-2019/situation-reports (accessed July 4, 2020).

[B120] Wu D. Koganti R. Lambe U. P. Yadavalli T. Nandi S. S. Shukla D. (2020). Vaccines and therapies in development for SARS-CoV-2 infections. *J. Clin. Med.* 9:1885. 10.3390/jcm9061885 32560227 PMC7355822

[B121] Wu P. Hao X. Lau E. Wong J. Y. Leung K. Wu J. T. (2020). Real-time tentative assessment of the epidemiological characteristics of novel coronavirus infections in Wuhan, China, as at 22 January 2020. *Eur. Commun. Dis. Bull.* 25:2000044. 10.2807/1560-7917.ES.2020.25.3.2000044 31992388 PMC6988272

[B122] Wu R. Wang L. Kuo H. D. Shannar A. Peter R. Chou P. J. (2020). An update on current therapeutic drugs treating SARS-COV-2. *Curr. Pharmacol. Rep.* 11 1–15. 10.1007/s40495-020-00216-7 32395418 PMC7211915

[B123] Xu H. Zhong L. Deng J. Peng J. Dan H. Zeng X. (2020). High expression of ACE2 receptor of 2019-nCoV on the epithelial cells of oral mucosa. *Intern. J. Oral Sci.* 12:8. 10.1038/s41368-020-0074-x 32094336 PMC7039956

[B124] Xu X. Han M. Li T. Sun W. Wang D. Fu B. (2020). Effective treatment of severe SARS-COV-2 patients with tocilizumab. *Proc. Natl. Acad. Sci. U.S.A.* 2020:202005615. 10.1073/pnas.2005615117 32350134 PMC7245089

[B125] Xue X. Yu H. Yang H. Xue F. Wu Z. Shen W. (2008). Structures of two Coronavirus main proteases: implications for substrate binding and antiviral drug design. *J. Virol.* 82 2515–2527. 10.1128/jvi.02114-07 18094151 PMC2258912

[B126] Yan L. Zhang H.-T. Goncalves J. Xiao Y. Wang M. Guo Y. (2020). Prediction of criticality in patients with severe Covid-19 infection using three clinical features: a machine learning-based prognostic model with clinical data in Wuhan. *medRxiv* [Preprint]. 10.1101/2020.02.27.20028027

[B127] Yang H. Yang M. Ding Y. Liu Y. Lou Z. Zhou Z. (2003). The crystal structures of severe acute respiratory syndrome virus main protease and its complex with an inhibitor. *Proc. Natl. Acad. Sci. U.S.A.* 100 13190–13195. 10.1073/pnas.1835675100 14585926 PMC263746

[B128] Yazdany J. Kim A. (2020). Use of Hydroxychloroquine and Chloroquine during the SARS-COV-2 pandemic: what every clinician should know. *Ann. Intern. Med.* 172 754–755. 10.7326/M20-1334 32232419 PMC7138336

[B129] Ye Q. Wang B. Mao J. (2020). The pathogenesis and treatment of the ‘Cytokine Storm’ in SARS-COV-2. *J. Infect.* 80 607–613. 10.1016/j.jinf.2020.03.037 32283152 PMC7194613

[B130] Zaki A. M. Sander V. B. Theo M. B. Albert D. M. E. O. Ron A. M. F. (2012). Isolation of a novel coronavirus from a man with pneumonia in Saudi Arabia. *N. Engl. J. Med.* 367 1814–1820. 10.1056/NEJMoa1211721 23075143

[B131] Zhang H.-T. Zhang J.-S. Zhang H.-H. Nan Y.-D. Zhao Y. Fu E.-Q. (2020). Automated detection and quantification of COVID-19 pneumonia: CT imaging analysis by a deep learning-based software. *Eur. J. Nuclear Med. Mol. Imag.* 47:9531. 10.1007/s00259-020-04953-1 32666395 PMC7358997

[B132] Zhang J. J. Dong X. Cao Y. Y. Ya-Dong Y. Yi-Bin Y. You-Qin Y. (2020). Clinical characteristics of 140 patients infected by SARS-CoV-2 in Wuhan, China. *Allergy* 75 1730–1741. 32077115 10.1111/all.14238

[B133] Zhang N. Li C. Hu Y. Li K. Liang J. Wang L. (2020). Current development of SARS-COV-2 diagnostics, vaccines and therapeutics. *Microb. Infect.* 22 231–235. 10.1016/j.micinf.2020.05.001 32387332 PMC7200352

[B134] Zhang Q. Wang Y. Qi C. Shen L. Li J. (2020). Clinical trial analysis of 2019-nCoV therapy registered in China. *J. Med. Virol.* 92 540–545. 10.1002/jmv.25733 32108352 PMC7228274

[B135] Zhang L. Lin D. Sun X. Curth U. Drosten C. Sauerhering L. (2020). Crystal structure of SARS-CoV-2 main protease provides a basis for design of improved α-ketoamide inhibitors. *Science*. 368 409–412. 10.1126/science.abb3405 32198291 PMC7164518

[B136] Zhang Z. Wang S. Tu X. Peng X. Huang Y. Wang L. (2020). A comparative study on the time to achieve negative nucleic acid testing and hospital stays between danoprevir and lopinavir/ritonavir in the treatment of patients with COVID-19. *J. Med. Virol.* 92 2631–2636. 10.1002/jmv.26141 32501538 PMC7300667

[B137] Zhao M. (2020). Cytokine storm and immunomodulatory therapy in SARS-COV-2: Role of chloroquine and anti-IL-6 monoclonal antibodies. *Intern. J. Antimicrob. Agents* 55:105982. 10.1016/j.ijantimicag.2020.105982 32305588 PMC7161506

[B138] Zhou M. Zhang X. Qu J. (2020). Coronavirus disease 2019 (SARS-COV-2): a clinical update. *Front. Med.* 14 126–135. 10.1007/s11684-020-0767-8 32240462 PMC7115348

[B139] Zhu F.-C. Guan X.-H. Li Y.-H. Huang J.-Y. Jiang T. Hou L.-H. (2020). Immunogenicity and safety of a recombinant adenovirus type-5-vectored COVID-19 vaccine in healthy adults aged 18 years or older: a randomised, double-blind, placebo-controlled, phase 2 trial. *Lancet* 396 479–488. 10.1016/s0140-6736(20)31605-6 32702299 PMC7836858

[B140] Zhuobing L. Li D. Gongqi C. Chaohui Z. Li X. Luo W. (2020). Clinical time features and chest imaging of 85 patients with SARS-COV-2 in Zhuhai, China. *Front. Med.* 7:209. 10.3389/fmed.2020.00209 32574321 PMC7225607

